# Institutionalizing and sustaining social change in health systems: the case of Uganda

**DOI:** 10.1093/heapol/czx066

**Published:** 2017-07-19

**Authors:** Jerald Hage, Joseph J Valadez

**Affiliations:** 1 Center for Innovation, University of Maryland, College Park, MD 20742, USA and; 2 Department of International Health, Liverpool School of Tropical Medicine, Pembroke Place, Liverpool, L3 5QA, UK

**Keywords:** Community health, health systems, institutionalization, learning, lot quality assurance sampling, maternal and child health sustainability, programme evaluation, sub-Saharan Africa, Uganda

## Abstract

The key to high impact health services is institutionalizing and sustaining programme evaluation. Uganda represents a success story in the use of a specific programme evaluation method: Lot Quality Assurance Sampling (LQAS). Institutionalization is defined by two C’s: competent programme evaluators and control mechanisms that effectively use evaluation data to improve health services. Sustainability means continued training and funding for the evaluation approach. Social science literature that researches institutionalization has emphasized ‘stability’, whereas in global health, the issue is determining how to improve the impact of services by ‘changing’ programmes. In Uganda, we measured the extent of the institutionalization and sustainability of evaluating programmes that produce change in nine districts sampled to represent three largely rural regions and varying levels of effective health programmes. We used the proportion of mothers with children aged 0–11 months who delivered in a health facility as the principal indicator to measure programme effectiveness. Interviews and focus groups were conducted among directors, evaluation supervisors, data collectors in the district health offices, and informant interviews conducted individually at the central government level. Seven of the nine districts demonstrated a high level of institutionalization of evaluation. The two others had only conducted one round of programme evaluation. When we control for the availability of health facilities, we find that the degree of institutionalization is moderately related to the prevalence of the delivery of a baby in a health facility. Evaluation was institutionalized at the central government level. Sustainability existed at both levels. Several measures indicate that lessons from the nine district case studies may be relevant to the 74 districts that had at least two rounds of programme evaluation. We note that there is an association between the evaluation data being used to change health services, and the four separate indicators being used to measure women's health and child survival services. We conclude that the two C’s (competent evaluators and control mechanisms) have been critical for sustaining programme evaluation in Uganda.

Key Messages

- Defines and quantifies the degree of institutionalization as measured by capacity of local health workers to carry out and use programme monitoring and evaluation (M&E), and provides a health care mechanism that supports the use of M&E to improve programmes.
- Documents sustainability of continued training and funding at the district level.
- Provides examples of learning from M&E results to change programmes and measures the impact of these changes on health care using four indicators.
- Reports on institutionalization and sustainability at the national level.
- Provides a critical lesson about the choice of LQAS data collectors.

## Introduction

Increasingly, leaders of developing countries and their donors are interested in understanding how to improve a population’s health status through high impact interventions (www.gatesfoundation.org). One method for achieving impact is through programme evaluation, even though the results may occur as incremental change, gradually over time. The current resource constraints in most major donor countries have led to declining support for health care and an increasing demand for developing countries to assume the costs of health care. This pressure highlights the importance of sustaining health care evaluations (USAID 2013a). For example, The United States Agency for International Development (USAID) has begun to reduce the size of its support for HIV/AIDS in Africa (Stash 2012). As a consequence, it has become more important to identify strategies that increase the potential for institutionalizing and sustaining programme evaluation, especially evaluations that lead to improved health services.

A good example of how to do this is found in Uganda, where Lot Quality Assurance Sampling (LQAS) (Valadez 1991) was used to empower (Robertson *et al*., 1997; Pagano and Valadez 2010) local managers to improve programme impact at a local level. Reports about health indicators do motivate organizational learning (Argote and Miron-Sepktor 2011; Valadez 2014b), i.e. using the information to make changes to health programmes where change is needed. In this study, we attempt to identify conditions at both local and central government levels that increase the acceptance of intervention changes that result in higher impacts.

Uganda represents an interesting case study for obtaining lessons about conditions that appear to increase the potential for institutionalizing and sustaining change through programme evaluation. Most primary health care interventions had been decentralized to the district government by the time this investigation began. Uganda has also had a relatively lengthy experience with this monitoring and evaluation (M&E) methodology, having been introduced to it in 2003 by the World Bank's Uganda AIDS Commission (UAC), funded through the Multi-Country AIDS Project (Jeffery *et al*., 2015; Crossland *et al*., 2015; Olanrewaju *et al*., 2015). Most projects share a common list of 59 indicators, representing one of the most widespread applications of programme evaluation with LQAS to date (Robertson and Valadez 2006).

The first section of this article reports the history of the institutionalization of LQAS programme evaluation within Uganda and the special conditions that appear to have facilitated its success in achieving high impact interventions. The second section focuses on our research design, the data collection methods for measuring the institutionalization, sustainability and learning that produced intervention changes in nine case studies. An important issue we consider is the extent to which one generalize from our case study findings to other districts in Uganda. We report our findings in the third section, while the Discussion section indicates lessons learned about institutionalizing and sustaining evaluation systems, and qualifications about the applicability of the case of Uganda to other developing countries.

## Institutionalizing LQAS programme evaluations in Uganda and facilitating societal conditions for its sustainability

**Figure 1.**
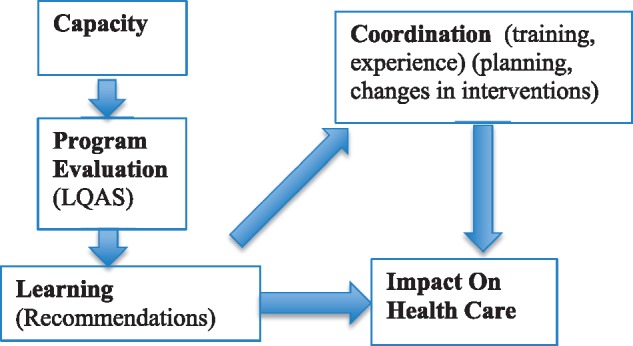
Institutionalization of programme evaluation to support health care

The twin concepts of institutionalization and sustainability have long been important ideas in the development literature, starting with concerns about maintenance of public works once built with international funding. In the social science literature, institutionalization is defined as clusters of norms (or behaviours) with strong but variable mechanisms of support and enforcement (Colyvas and Powell 2006; Rueschemeyer 2009). Few empirical studies of the process of institutionalization exist. One recent study in a Armenia cited guidelines, an office, skilled staff and internal finances as measures of institutionalization, but it ignored issues such as the variety of staff, their level of experience and, most critically, measures of coordination and control or enforcement of the guidelines and human resource policies (Yoder 2011). This approach to the study of institutionalization focuses on mechanisms to stabilize a norm or a practice. However, once the focus shifts to the issue of the institutionalization of programme evaluation, the definition of institutionalization should involve ‘change’ since programmes should use data to improve themselves. Specifically, institutionalization concerns whether recommendations about changes in health services are accepted and whether the changes lead to changes in health behaviour. Despite this shift from questions of ‘stability’ in rules to ‘changes’ in procedures, the same two C’s themes in the institutional literature remain: capacity (training and experience) and control/coordination (incorporating recommendations into planning and changing interventions). This shift to change underscores the importance of examining learning resulting from using evaluation data to change interventions; this data use is a critical intervening variable (see Figure 1) (Argote and Miron-Sepktor 2011). ‘Sustainability’ means, in operational terms, the continued training of programme evaluators and the funding of evaluations.

**Figure 2.**
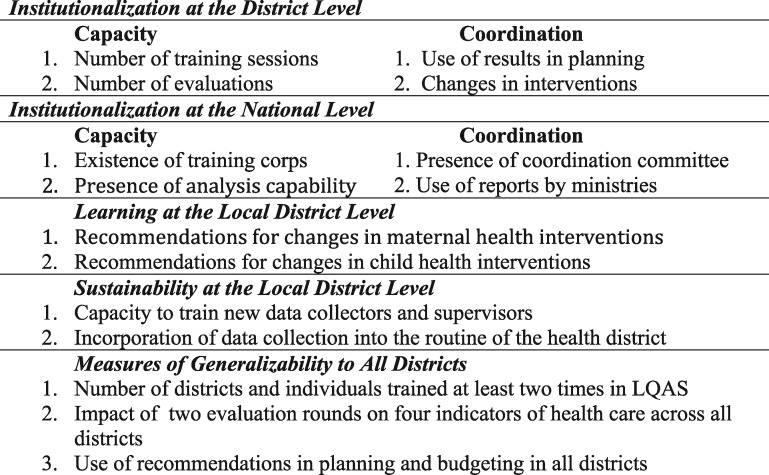
Concepts and measures of institutionalization

Although institutionalization and sustainability are predominant concerns of donors working in low resource settings, little has been written about learning. Learning is the essential reason that programme evaluations are undertaken. The data are intended to be used to improve programmes. A great advantage of studying the institutionalization process of the LQAS methodology at the district level is the ability to examine whether there is a direct link between the district level presentation of results, the making of recommendations to correct particular deficiencies, and the adoption of strategic and tactical changes in health programmes. This is the central argument about how programme evaluations can contribute to making high impact interventions. It is often assumed that once locally relevant information of poor performance is detected by local managers, they will change their strategies and adopt more effective interventions, but this is not enough. Using data requires understanding the problems it signals and the reasons they occur; also it requires the active use of the data to modify the programme. Together these steps constitute ‘learning’. This link of data capture to its use is why ‘learning’ should be included among the major M&E concepts (Argote and Miron-Sepktor 2011). Furthermore, the best test of learning is assessing an attempted improvement to learn if it achieves the expected impact (see Figure 2) (Valadez *et al*., 2005).

Consistent with the importance of measuring capacity to conduct programme evaluations, Uganda has had considerable experience with the LQAS methodology. As noted earlier, it was introduced in 2003 to evaluate HIV interventions. That project built LQAS capacity in multiple governmental and civil society organizations in addition to the UAC. During 2009, when the STAR-E programme began, USAID required that all of its major projects [Strengthening TB and HIV/AIDS Responses-Eastern Region (STAR-E), Strengthening TB and HIV/AIDS Responses-East Central Region (STAR-EC), Strengthening TB and HIV/AIDS Responses-South West Region (STAR-SW)] use the LQAS evaluation methodology and the same indicators. Later, this mandate was extended to other USAID projects [**STRIDES** for Family Health Strengthening Uganda s (STRIDES), National Response for Implementation of Services (SUNRISE), Northern Uganda-Health Integration to Enhance Services (NU-HITES), Civil Society Fund (CSF), Stop Malaria Project (SMP)]; it was then supported by UNICEF and the Department for International Development (Valadez *et al*. 2012). The 59 indicators covered the following areas: (1) reproductive health and family planning; (2) child health; (3) malaria control and prevention; (4) sexually transmitted diseases prevention including HIV counselling; (5) tuberculosis control; (6) water and sanitation; (7) nutrition; (8) orphans and other vulnerable children; and (9) education. Four distinct organizations manage the seven projects (see the Supplementary material for details). Thus, Uganda has had extensive experience in programme evaluation at the district level. However, the districts vary considerably in the number of times the evaluation method has been conducted (see Supplementary Table S1 for the nine case studies). Therefore, it is important to study the district level to understand the impact of the evaluations on health programmes.

Two important distinctive characteristics of Uganda that potentially facilitate both the institutionalization and sustainability of the LQAS methodology are: (1) the decentralization of most government services to the local government level, and (2) the still relatively high level of social capital (Portes 1998) prevailing, at least, in the rural areas. Decentralization of health services has meant that decisions made by the district health officers could be implemented. In other words, both the power to use data and the programme evaluation were decentralized, meaning that local individuals could use recommendations to change programmes. The importance of social capital at the local level is that recommendations are more likely to be implemented. Social capital can be measured by the aid individuals give to each other, as well as the provision of public goods, e.g. transportation to health clinics. These characteristics are important, because together they allow for mobilization of the local population when the results of the LQAS evaluation are presented. It also means that greater attendance of the meetings at which findings are presented, by those for whom the results are relevant, causes more effective problem solving, facilitating the implementation of changes, especially when they require changes in local programme tactics (Valadez *et al*. 2005). Although these characteristics may make the transfer of lessons learned in Uganda to other countries more difficult, they also have implications for the research methods we used. 

## Methods

Uganda provides an opportunity for in-depth study of how to measure the institutionalization and sustainability of LQAS methodology in health care as well as the learning that results. Given our concern with understanding whether programme evaluation is producing high impacts, we selected nine districts in three different regions, three districts each in the eastern, western and southwestern regions. We first based our selection on the region, because we assumed that regional differences were associated with institutionalization and sustainability. By selecting diverse districts within each region, we can assess the effect of demographic and spatial differences more directly. Rural areas were selected, as they have the poorest populations with the greatest need for health services and frequently lack adequate medical facilities. Time limitations prevented adding more regions or districts within the regions. The northern region was not included, because the NU-HITES project had just started. The central and east-central regions were not included because of the higher rate of urbanization (see the Supplementary material for more detail on the research design and the selection of districts).

Since responsibility for managing most components of the health system is decentralized in Uganda, the district is the most important level for examining whether institutionalization and sustainability of programme evaluation exist. Another reason for this focus is that the LQAS programme evaluation methodology examines the impact of interventions at the sub-district level and makes recommendations at this level; it therefore allows us to measure local learning. Our focus on the local level is in contrast to much of the current literature that discusses institutionalization and sustainability on the central government level (Xaxas and Vogel 2007; Stash 2012; USAID 2013b). The central government is not irrelevant. It needs to be considered, but the meaning of institutionalization and sustainability and how these concepts are measured differ (see Figure 2).

As district governments are organizations our research design takes account of this fact by conducting interviews at three distinct organizational levels in each district: (1) directors; (2) supervisors of programme evaluation; and (3) data collectors. At level 1, two directors were interviewed: the district local health officer (DHO) about maternal health service, and the Assistant DHO about children’s health. At levels 2 and 3, we organized two large focus groups; time constraints prevented us from interviewing three to five members in each group separately. Additional data were obtained by asking each supervisor and data collector to fill out a short survey about their position within health districts, their responsibilities, and their level of LQAS training (Valadez *et al.* 2007). At the national level, we conducted informant interviews.

We constructed the interview tools to obtain information from two different sources for each major concept. For example, the DHO was interviewed about their use of LQAS results to modify maternal health services on two indicators (ANC1+ and delivery in health facility) while the Assistant DHO was asked primarily about improvements related to two child health indicators (treatment seeking for a sick child in a health facility (HF) and up-to-date immunizations of children 12–23 months of age).

## Measurement indices

At the local government level, institutionalization is measured with two indices, one for each of the two C’s: (1) ‘capacity’ because of training and experience, and (2) ‘control/coordination’ resulting from changes in interventions derived from using the evaluation data and ensuing recommendations (see Figure 2). We use similar indices at the central government level. A neglected consideration, the amount of learning resulting from programme evaluation is measured by recommendations for changes in the intervention strategies and tactics and their impacts on health care. Sustainability is measured by the capacity to continue training and funding programme evaluation.

The first C (capacity of the evaluation team) is measured by the level of training and experience of the district LQAS data collectors and their supervisors. The central reasoning is that as the number of times they participate in LQAS training and in data collection increases, the more likely they are to be competent and capable of continuing if project funding is no longer available. In addition, the supervisors were asked to evaluate the competence of their data collectors. Following this question, we asked the supervisors if they thought that the data collectors were capable of collecting data without supervision (Hage 1974; Beckworth 2015). For the supervisors, we asked what their own duties were as a check on their knowledge. We also asked both the DHO and the Assistant DHO about their exposure to the LQAS system and whether they had participated in any phase of the process from planning through to the feedback of the results (for details about scoring the indices, see the Supplementary material).

The concept of control/coordination is complex. We measured two important components: the use of recommendations in planning and budgeting, and the use of recommendations to change intervention strategies. As our method collected data from three different levels of the district health office, we were able to cross-check whether recommendations actually led to changes in intervention strategies. We documented changes in strategies resulting from LQAS data that are associated with increases in maternal and child health indicators.

We also measured the amount of training and control/coordination to assess the degree of institutionalization at the central government level. Two sets of skills at this level were measured: the training of National LQAS Facilitators (NLFs) who train supervisors and data collectors, and people trained in LQAS data analysis and data use at the various ministries. Only raw numbers of personnel are reported because measures of their experience were not available. However, our data capture the diversity of skills needed for institutionalization.

We also measure national control/coordination at the central government by documenting the extent to which ministry officials report their use of LQAS results. A second measure is whether an M&E coordination group has been established in the central government. This information is further evidence of increased control/coordination.

The essence of organizational learning is the production of recommendations for programme improvement (Figure 1). Two categories of recommendations were used to measure learning; those related to maternal health programmes and to children’s health. Examples are provided in the case studies. Consistent with the organizational literature, we also measure learning by documenting changes in output indicators of health care (Argote and Minor-Sepktor 2011).

For donors and governments, a priority concept is sustainability. Although a set of skills may have been institutionalized, the issue remains whether these skills will continue to be utilized at a relatively low cost. The latter is an important constraint and can impede the institutionalization process. Governments, such as Uganda’s, have limited resources and therefore, sustainability also requires low cost strategies. The first sustainability measure is continuation of staff training; this assessment includes whether current supervisors and data collectors are capable of training their replacements and personnel in other districts. The second measure is documenting whether data collection and analysis are integrated into the regular work routines of the data collectors and supervisors. We assessed integration of LQAS into the routine work of data collectors and supervisors at all three levels of the district government. In the central government, we also assessed sustainability in various ministries by asking whether they are prepared to continue supporting the LQAS strategy.

Since the nine districts represent a small fraction of the 112 districts in Uganda, our design should allow for generalizing conclusions from the rich data in the nine cases studies to the entire country. Three distinct measures were used to assess generalizability. First, the amount of training in programme evaluation should not vary among districts and can be assessed using administrative records. Second, improvements to health care (two for maternal health and two for child health) we hypothesize are associated with exposure to two or more rounds of programme evaluation. This analysis infers that the use of recommendations to change interventions is a measure of learning; this is an analysis recommended in the organizational literature (Agrote and Minor-Sepktor 2011). Finally, we carried out a survey to measure the use of recommendations in planning and budgeting.

## Research findings

The institutionalization of programme evaluation using LQAS (or any other approach) is not a binary condition: presence or absence. Rather it is a process that comes in degrees. By measuring institutionalization in this way, we can understand how to further advance this process. Also, the process of institutionalization varies at two distinct levels of analysis: (1) in districts; and (2) in the central government. The findings on institutionalization for these two levels are reported first, followed by sustainability; we conclude with our assessment of the generalizability of findings. We provide examples of district level learning together with the discussion of local institutionalization.

### Local district level

**Figure 3.**
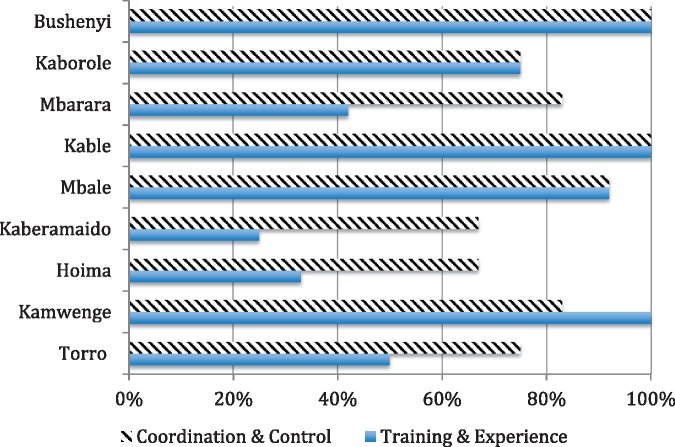
Degree of institutionalization based on training and experience, and coordination and control: percentages computed on a total of 12 points

The first index of institutionalization measures capacity (training and experience,) which scored high in five districts with ≥75% (see Figure 3). Three districts (Bushenyi, Kabale and Kamwenge) scored 100% on this index ($\overset{-}{x}$ = 68.5%, SD = 31.2%). Admittedly, the major determinant of these scores is the number of times the district participated in LQAS data collection and hand tabulations (see the Supplementary material). Districts having four or more LQAS rounds did not receive extra credit since our experience indicates that three applications are sufficient to inculcate the skills needed for routine use. We should note that consistently individuals who had multiple rounds of experience also reported that they received refresher training for each round. No additional weight was given for this experience, as refresher training was so closely associated with having participated in multiple LQAS rounds.

The second index of institutionalization measures control/coordination, which scored ≥75% in seven districts (Figure 3). The only exceptions are Hoima and Kameramaido ($\overset{-}{x}$ = 82.4%, SD = 12.7%). In other words, the institutional control/coordination index is higher than the capacity index.

Some examples of how the districts changed their intervention strategies and tactics, and thereby demonstrated learning, are as follows. The respondents in Kamwenge stated that they used the results to secure additional funding and mentioned, in particular, attempts to increase their rates of immunization and male circumcision. Bushenyi was concerned by their low rate of ANC4+, and started working with the village health technicians (VHTs) to encourage mothers to visit the HF. The VHTs were instructed to visit houses to emphasize exclusive breastfeeding. Also they trained the VHTs to train mothers to recognize danger signs and political leaders to promote immunization. Finally, district management teams were explicit about their intention to build a new health facility each year and train the staff for it on the basis of the LQAS results. Mbale also used the LQAS results to plan infra-structure. Following many of the same procedures as Bushenyi, in Mbale the VHTs escorted mothers to the HF. They also encouraged fathers to transport mothers. In sum, the DHO in Mbale thought that LQAS pushed his team to take more responsibility for their district.

Although Kaberamaido scored relatively low for control/coordination, the respondents reported using LQAS data to improve coverage with appropriate sanitation. The scores on breastfeeding were used in Tororo to inform changes in the prevalence for this array of interventions. This district also illustrated a new challenge; their strategic changes using LQAS data produced such a large increase in demand for HF services that patients complained of longer queues and waiting times. However, new problems surfaced such as the absence and/or failure of electrical supply at the HF, which led to women leaving the facility prior to the delivery of their babies. Mbarara used the LQAS results to improve family planning and immunization services. Interestingly, district managers concluded they had not been making sufficient effort to reach the susceptible population. As a result, they improved education of mothers and encouraged fathers to take women and children to the HF. To improve service coverage in Kabale, the DHO had their VHTs make house-to-house visits to explain that health services at the HF were free. They integrated promotion of immunization with other health services to improve their score on this indicator.

At the beginning of this report, we assumed there was a relationship between the degree of institutionalization and the effectiveness of health service delivery (Figure 1). This idea can be tested by examining the relationship between these two variables controlling for the availability of HF, which as we have seen in Tororo is an important constraint. The two scores on institutionalization in (Figure 3) were added together and assigned ranks. The same was done for the frequency of using a HF for delivery relative to the availability of HFs. The Spearman’s Rho (Rho = 0.45) correlation of these two ranks indicates the relationship is moderately strong. Given the above data on the number of examples about how recommendations were used, we have more confidence about the generalizability of this correlation.

### National level

National level managers require different kinds of skills than those that are needed by district health officers, data collectors and supervisors. The first asset is a cohort of national evaluators and trainers-of-trainers. A group of 71 NLFs were trained by 2014. They have become critical for sustaining the LQAS method in Uganda. The second resource is a national LQAS database that combines all the individual LQAS databases; Uganda developed a single ‘super database’ integrating data from all participating districts from 2003 through the present time. The Ministry of Local Government (MoLG) agreed to house it for future generations to use and add to. To date 15 individuals have received statistical training and can use these data for preparing reports for central government and publications. This asset represents a potential source of future learning and is one way in which learning can be sustained at a national level. Relative to coordination, in an interview with the national coordinator of STAR-E LQAS, we learned that the Ministry of Gender, Labour and Social Development has found the data to be quite useful and was establishing a coordinating committee at the time of this study.

### Sustainability

The major question is whether LQAS as a strategy of social change can be sustained. The first measure of sustainability is whether the programme evaluation skills can be transferred to those districts with only one or two rounds of data collection and even more to those districts with no rounds. All the data collectors and supervisors believed that they were trained well enough so they could train others. In this context, the NLFs are a vital resource for transferring knowledge.

The second issue is whether funding of the programme evaluations can be continued. In the interviews with the DHOs, Assistant DHOs and the focus groups, we asked whether the data collection could be integrated into the on-going work of the supervisors and the data collectors. There was nearly complete consensus that this could be done. From the perspective of the DHOs and their Assistants a typical response about continuing to use LQAS was: ‘Why Not?’ And some interviewees indicated that the district was having active discussions about how this might be accomplished. Quite surprisingly, few mentioned the loss of the per diem that they received when collecting data as part of the current arrangement with STAR-E LQAS. One component of data collection that requires some expenditure of funds, which was repeatedly mentioned, is the adequacy of transportation. Therefore, if programme evaluation is to be sustained, this is a major challenge, not only because of the necessity of visiting villages that are remote but also because the equipment provided is sometimes inadequate.

Sustainability at the national level should occur because Mr. Mutabwire, Director of Local Government Administration (MoLG), is a champion of this methodology. Local ownership in most of the districts is complemented by national ownership (USAID 2013a) The Director formed and chairs a technical working group focused on the sustainability of LQAS and even its expansion to include more indicators in sectors of concern. He stressed the importance of schooling and the failure of the public sector to provide adequate education.

### Generalizing from the nine districts to all districts

Although the nine case studies contain a great deal of rich information, not all of which is reported here, the issue remains whether the findings based on these case studies can be generalized to other districts. Three measures indicate that they can be. First, after 6 years of the STAR E-LQAS project, a total of 1388 people were trained by 2013 to use the LQAS methodology. However, by 2014, only 66% of the districts had had two rounds of LQAS. Using this as the standard of capacity, we estimate that 867 of 1388 supervisors and data collectors had this minimum experience.

Perhaps the most important measure of the ability to generalize from the nine case studies to all districts that have had exposure to programme evaluation is the replication of the analysis above where the degree of institutionalization was found to be related to the impact on health care. Table 1 reports the average effectiveness increase on four separate measures of health care for 74 participating districts, which includes seven of the nine case study districts that had two or more rounds of programme evaluation between 2009 and 2014. Gains of 9-10% were achieved in three of the four indicators. In 43 districts, the ‘Service Performance Assessment and Improvement’ (SPAI) method for analyzing the causes of various failures in implementation and how they might be improved was used. The issue is whether this process increased the effectiveness of the LQAS methodology and reinforced the need to make changes in the interventions. Clearly, the collective learning in SPAI (5 days) and SPAI LITE (2–3 days) reinforced the learning that comes from the hand tabulations presented in the preliminary reports of LQAS. Further learning occurred because STAR-E LQAS held one national meeting and several regional meetings about how to improve the interventions using the LQAS results.

**Table 1.** The amount of learning in maternal and child health programmes: percent increase of four indictors

Average increase in percentage across districts			
LQAS indicator	All districts	LQAS only districts^a^	LQAS + SPAI districts^b^
4+ antenatal visits	9.7%	7.7%	11.0%
Delivery in a HF	10.1%	7.0%	12.1%
Child immunization	9.0%	5.4%	11.6%
Sick child visits to a HF	3.8%	2.0%	5.1%
Number of districts implementing	74^c^	31	43^a^

a Includes LQAS training, data collection, and hand tabulations of results.

b Includes both SPAI and SPAI LITE. Seven districts had two interventions; hence the number of districts is 43 and not 50.

c 12 districts out of the 86 involved in LQAS in the period of 2009–14 did not have a second LQAS survey.

On the basis of a survey in 2013, 53% of the districts participating in an LQAS round reported that they used the recommendations from the programme evaluation in planning and 47% used them in budgeting. Again, these estimates are conservative because only 61% of the participating districts returned their report. The intensive case study of the nine districts, reported above, suggests that these percentages probably should be higher since seven of the districts, six of which had two rounds of LQAS surveys, scored 75% or more on the use of recommendations, and several did not return their survey.

## Discussion

Considerable evidence exists that social change in health care in Uganda has been institutionalized as a function of the LQAS programme evaluation method. Of the two indices measuring institutionalization, the control/coordination index had the highest scores. This important finding means that seven of the nine districts are learning, as measured by programme changes taking place using LQAS results for planning and budgeting. The two districts that scored lower on both indices, Hoima and Kaberamaido, provide an instructive contrast. Kaberamaido, even without a second round of data, scored a big success in increasing coverage with improved sanitation in this district, and this result empowered the supervisors and data collectors to continue using LQAS (an additional round was scheduled after this study). Also, they learned another benefit of using LQAS results; namely, they were able to obtain additional funding from the CSF to address problematic HIV/AIDS interventions and resolve deficiencies. This benefit helps to explain why a second round of data collection can be so crucial for institutionalizing LQAS. Districts may need the first-hand experience to learn that LQAS can aid them to obtain resources recurrently, thereby, justifying its inclusion in the district planning process. Although in contrast to Kabermaido, Hoima has made a number of attempts to use the LQAS data—an observation based on the availability of reports—they did not have any measure of whether their changes in strategies and tactics had a pay-off. This district was the only one not interested in having another round of LQAS. One implication of this tale of two districts is the importance of ensuring that there are always at least two rounds of LQAS that are supported so that districts can verify that changes in indicators demonstrate the efficacy of this methodology and provide the data collectors and supervisors with a sense of empowerment.

This finding also suggests that requiring three rounds of LQAS for measuring the capacity index of institutionalization is not always necessary; two rounds appear to be sufficient. However, it may be that during the third round the process is further reinforced. In addition to the institutionalization at the local level, institutionalization also occurs at the central government level. The institutionalization of social change at the national level seems assured because the MoLG has taken responsibility for creating a coordinating committee.

Our analyses indicate that two rounds of programme evaluation produce improved health care, at least for the four indicators examined. The many learning examples in the case studies suggest that there is a causal connection. At the same time, future research should explore why some districts with relatively lower availability of HF are able to have more deliveries and vice-versa.

It is important to recognize that not all the credit for programme improvement can be given to the institutionalization of programme evaluations. Learning also occurred for other reasons. The MoH changed its policy and instituted the development and training of VHTs. Most of the nine districts reported on this important change and its consequences for improving the effectiveness of health services. When LQAS reports were made, frequently a solution for a problem was the decision to use VHTs to improve social services and in particular to support maternal and child health services. They became important advocates for changes in the intervention strategies and were also effective in implementing some of these changes. The MoH also held meetings to provide information about how to improve services.

The evaluations are sustainable because there is a trained cadre of individuals at both the local and the national level that can teach others LQAS methods. In the beginning of the project, STAR-E LQAS made the critical decision to recruit individuals who would be trained from district level staff. This is an important lesson for other countries as well as for international donors. The future costs for the LQAS should be relatively low once the work is incorporated into the routines of the district offices; other countries have taken this important step (Valadez and Devkota 2002; Valadez *et al*. 2014a).

How easy is it to extrapolate these results to other countries in sub-Saharan Africa? Certain conditions exist that have increased the likelihood that a project would become institutionalized and sustainable; such conditions may not exist elsewhere. The locus of decision-making in local governments is one special condition that resulted in more success in Uganda. Decentralization results in higher local participation when the preliminary results are obtained. It also strengthens local social capital, which increases participation when recommendations are discussed. Due to the wide participation at these meetings, there is better problem solving and a greater commitment to decisions that are made about how to improve the interventions. Another key condition that has increased commitment and sustainability is the choice of full-time employees of the local government to be data collectors and supervisors. This choice has meant that trained personnel do not disappear when the STAR E-LQAS project ends. They remain to sustain the institutionalization process and transfer knowledge to new districts and new officials. However, we note that the Ugandan government at the central level is more decentralized than many other sub-Saharan African countries. Thus, the MoLG could become a critical champion that is capable of providing more opportunities to institutionalize M&E than the MoH is able to muster.

This article provides a methodology for studying the institutionalization and sustainability of social change via programme evaluation. The indices are complex and capture many of the ideas that are contained in the organizational literature. However, we have emphasized change rather than stability, making the definitions and the measures more appropriate for developing countries concerned with improving their health systems and their societies.

## Supplementary Data

Supplementary data are available at *Health Policy and Planning* online.

## Supplementary Material

Supplementary Data
