# Supplementary material for: Institutionalizing and sustaining social change in health systems: the case of Uganda
Source: Health Policy Plan. 2017 Jul 19;32(9):1248–55. doi: 10.1093/heapol/czx066 (PMC5886222; doi:10.1093/heapol/czx066)
Supplement: Supplementary Data [file czx066_supp_appendix_6_june_2016.docx]

**Appendix**

**The LQAS Projects in Uganda**

USAID supported a set of three health projects in Uganda during 2009-15 collectively named “Strengthening TB and HIV & AIDS Responses” (STAR). The three projects work independently in three regions (East, East Central, and South West) bearing these locations in their name. STAR-E (East) included a sub-project named STAR E-LQAS; it is collaboration between Management Sciences for Health (MSH) and the Liverpool School of Tropical Medicine (LSTM). Its mandate from USAID included training and supervision to district and central government bodies to use LQAS to monitor health indicators, and to support Uganda to institutionalize this method. It builds on Uganda’s previous exposure to this methodology.

The USAID projects using LQAS were: Strengthening TB and HIV& AIDS Responses in East, East Central and South West Uganda (STAR-E, STAR-EC, STAR-SW); STRIDES for Family Health (STRIDES); Northern Uganda Health Integration for Enhanced Services (NU-HITES); USAID’s Orphans and Vulnerable Children Project (SUNRISE); Civil Society Fund (CSF); and the Stop Malaria Project (SMP).

The MSH/LSTM partnership or STAR-E LQAS provided technical assistance in the use of the LQAS methodology to these partners. In addition, to these organizations, several international non-government organizations (INGOs), and local non-government organizations (NGOs) also developed a capacity to use LQAS; they include AfriCare, AidChild, and the Program for Accessible Health Communications and Education (PACE), and The AIDS Support Organization (TASO). Further support came from UNICEF working with Child Fund International and LSTM to use LQAS together with other management methods to support decision-making for Integrated Community Case Management of child health in 21 Districts; and lastly, UKAID through DfID supported the assessment of the effectiveness of Results Based Financing in the Acholi and Lango sub-regions of Uganda while building district capacity to use LQAS. In 2009 the STAR-E LQAS project supported 10 districts; by 2014 it supported 86 of 112 districts to use LQAS at least once; 74 (66%) have experienced at least two LQAS surveys. Therefore, Uganda represents a massive effort and a good test case of the effectiveness of LQAS to produce change(Beckworth et al., 2015, Valadez et al., 2014b). Some of these changes have already been documented (Valadez et al., 2014b, Crossland et al., 2015, Jeffery et al., 2015, Olanrewaju et al., 2015).

**What LQAS Is**

Uganda used standard LQAS procedures (Robertson et al., 1997, Valadez, 1991). LQAS is an established analysis technique (Robertson and Valadez, 2006) originally developed as a classification method for industrial quality control during the 1920s and adapted to health sciences in the mid-1980s (Valadez, 1991) to classify management units, referred to as supervision areas (SA) according to a performance target. In Uganda sub-district areas, usually counties or sub-counties are the SA. In each SA, a sample of “n” individuals is assessed. A “d” is selected that determines the cut-off number below which the area is classified as low-performance for a specified indicator. The decision rule “d” depends on the sample size, thresholds for classifying high and low performance, and selection of two misclassification errors, namely, the risk of misclassifying an SA with very low coverage as high (β error), and the risk of misclassifying an SA with high coverage as low (α error). The Ministry of Health or the donor set the upper threshold, “pU”, for identifying acceptably performing SA while low performers, “pL“, are often 30% lower(Pagano and Valadez, 2010). In our surveys, n=19 villages were typically selected in each SA. The SA sample size, n=19, ensures that α and β errors do not exceed 10% for all “pU”, and all corresponding values of “pL” that are 30 percentage points less than “pU” (Valadez et al., 2007), as is the case for this assessment. SA with pL < p <pU are also classified. A high classification is more likely the nearer p is to pU (and vice versa for pL); p located in the middle of this so-called grey area has an approximately equal chance of being classified in the high or low category.

**Detail on Research Design**

Seven DHOs and six Assistant DHOs participated in the study. The two focus groups included the participation of 31 LQAS supervisors and 38 data collectors. The scores in each of the nine districts in the three regions and their level of experience with LQAS as a method of program evaluation is contained in Table 1A.

**Scoring of Indices of Institutionalization**

The index of capacity has a maximum score of 12 points. For each year the DHO, Assistant DHO, supervisors and/or staff participated in data collection, one point was awarded up to a maximum of three. For the two district health managers, the DHO and Assistant DHO, the key issue was did they receive reports with feedback of the results, and not whether they had been trained in LQAS data collection procedures. If one or another of these managers or their substitutes was missing or if they were new and had not received any reports, a zero was assigned because they had had no exposure to LQAS, otherwise they received a one. As regards supervisors and the data collectors we found little variation among districts because most of them had participated in all rounds of data collection since 2009. A few individuals had also been involved in the earlier rounds prior to 2009 in the HIV/AIDS project supported by the World Bank.

The index of control/coordination is much more complex but again the maximum score is 12 points. We considered several options when constructing the coordination index since coordination can be accomplished via a number of mechanisms (Hage, 1974): use of manuals, quality controls, changes in intervention strategies, and the use of LQAS evaluations in planning or budgeting. Unlike the previous index that counts frequencies, this second index measures presence or absence of LQAS data during 2012 or 2013. We were not able to quantify how much change in intervention strategies/tactics took place or how important LQAS was for planning or budgeting. These are problems for future research. In measuring data use in planning and budgeting, there were some recall problems in one district, especially among the data collectors. Wherever possible the answers were checked against reports to be certain that an accurate assessment was made. Again, the index has four indicators: (1) use of the standard LQAS training manuals; (2) quality of data collection control; (3) report of the use of LQAS results to change or improve health program strategies; and (4) the report of the use of LQAS results in planning and budgeting by the DHOs and the Assistant DHOs. One point is given if the supervisors report the presence of a manual used for supervision and one point if the data collectors also report they had a manual. Two points were assigned to indicator 2 as there are two different ways for supervisors to check data quality by the supervisors: checking the tabulations and revisiting sampled homes to verify that the data had been collected and was correct Indicators 3 and 4 were worth four points each. The much higher weight for these two indicators reflects the importance of changing the strategies and tactics resulting from learning from recommendations and using the data to improve health programs through the district planning process and allocating resources in the district budget for this purpose. Both indicators are essential to any concept of institutionalization since they deal most directly with the ideas of control and coordination and yet are absent from previous discussions (Coylvas, 2006, Rueschemeyer, 2009, Yoder, 2011). See Table 2A for a summary of the scoring of both indices.

**Note about the Research Design**

The selection criteria for the regions and districts within them were as follows. The central region was excluded because of the high rate of urbanization and development. The northern region was excluded because a major USAID project, NU-HITES, had started in 2012, less than one year before this 2013 study. Therefore, evaluating institutionalization and sustainability was premature. At the time other regions such as Nile or northeast regions were not priorities. The western, southwestern, and eastern regions remained available for study.

The choice of the district was designed to maximize variation within a region. Table 1A indicates that despite the major differences among regions, there is also considerable variation within them as well. Although Table 1A uses data collected in 2012, four of the selected districts also collected data in 2013. The five exceptions are Kabarole, Mbarara and Tororo, whose implementing partner, Sunrise-OVC, did not have enough money to fund another round of data collection, and Hoima had funding for only one year from the CSF. The other exception is Kaberamaido, where the next round of LQAS was scheduled for 2014. Indeed, 20 out of the 72 districts reported in Table 1A did not have funding in 2013.

Another consideration in the research design was the possibility of major variations existing across the sub-districts. The LQAS system of sampling households in villages is designed to capture this distinctive form of variation and signal where specific problems exist. Both the data collectors and the supervisors were asked to report if significant differences existed within parishes or sub-counties in the areas in which they worked. The reasoning behind this was to better identify locally the important challenges to overcome and increase service delivery coverage.

This research design, a comparative case study, uses a purposive sample of districts. This approach is justified as we intended to assess linkages between measures of health service coverage and the districts’ use of LQAS for program planning. This comparative case method design, in the jargon of social research, sampled districts on the dependent variable. With this design, we assume that different levels of LQAS institutionalization can explain variation in percent of births in health facilities (Table 1A).

The data collection for this study consists of two in-depth interviews and two focus groups at the district level. Our basic principle was to obtain information from two different sources for each major index or sub-index (Table 2A). For example, the DHO was interviewed about learning relative to two separate indicators of safe motherhood (antenatal care visits and delivery in a health facility), while the Assistant DHO was asked primarily about learning relative to two indicators of child health (taking a sick child to a health facility and immunization). In some districts, the DHO or the Assistant DHO was absent and in others they were too busy preparing reports, participating in meetings or absent on trips. Wherever possible we conducted telephone interviews when these managers were not present on the day of our visit (Table 3A). Also, at times, knowledgeable substitutes (health educators) were found to ask the same battery of questions. In addition, wherever possible, documentation of the impact of LQAS on planning was secured to crosscheck and supplement the responses received in the interviews.

Separately the supervisors and the data collectors (Table 4A) participated in focus groups about various challenges and strategies for sustainability. Again, both were asked many of the same questions to obtain a crosscheck on the information. We used focus groups because there was not enough time to interview the three to five members in each group separately. Individual data was obtained by asking each supervisor and each data collector to filled out a short survey about their position within the health district, their responsibilities, and their level of LQAS training. The classifications of their major work responsibilities are contained in Table 4A which shows that the data collectors and the supervisors tended to work either in the health sector or in community development.

We also ask district level informants questions about how they interfaced with the central government in the use of LQAS data. We did this to assess their alignment with the decentralized model of the Ugandan government. We also conducted several interviews at the central government level to obtain some additional information about the challenges of sustainability. The following section contains the data collection instruments

**Interview and Focus Group Discussion Tools**

**Interview Questions Used to Measures of Institutionalization of LQAS: Training and Experience, Coordination and Control**

**Training and Experience**

***Data Collectors***

1. When did you receive your basic LQAS training? year: _______ year: _______

Which areas did you assess: _____

Reproductive health Tuberculosis

Child health Sexually transmitted diseases

Nutrition Sanitation

Malaria Other (please specify)

2. Circle each year in which you participated in data collection with LQAS:

2013 2012 2011 2010 2009

3. Have you had any LQAS refresher training? Yes/No When?

***Supervisors***

1. What are your duties as a supervisor?

2. When did you receive your supervisory training? year: _______ year: _______

Which areas did you assess: _____

Reproductive health Tuberculosis

Child health Sexually transmitted diseases

Nutrition Sanitation

Malaria Other (please specify)

3. Circle each year in which you participated in data collection with LQAS:

2013 2012 2011 2010 2009

3. Have you had any LQAS refresher training in supervision? When

4. In your opinion how competent are the LQAS data collectors that you supervised? If good, Could they work without supervision in the next round of data collection?

***DHO and Assistant DHO***

1. Please indicate the year you were first exposed to LQAS?

1a. What was the level of exposure? 1b. Did you receive any training in LQAS?

**Coordination: Manuals, Quality Control, and Planning**

***Data Collectors:***

1. Are there manuals available that you can refer to when collecting data?

***Supervisors:***

1. Is there a manual with clear guidelines for the supervision of LQAS?

2. What kinds of checks have you developed to verify the quality of the data?

***DHO and Assistant DHO***

1. Have you used LQAS results for making decisions about program improvement after any of these reports were received? If yes, in what year and how were they used how and when? If not what are the underlying cases that deterred you from not using?

**Measures of Challenges to Institutionalization**

**Data Collectors**

1. It is my understanding that you have multiple responsibilities. Please indicate what your duties are in this district. How much time do you spent in these activities?

2. Did you encounter any problems in collecting the data?

2a. If yes, what were they?

2b. Do you have suggestions for how these problems might be solved?

2c. Does collecting the data across 59 indicators present any special problems?

**Supervisors**

1. It is my understanding that you have multiple responsibilities. Please indicate what your duties are in this district. How much time do you spent in these activities?

2. What are the obstacles or problems that you face in your supervision work?

2a If yes, what are these?

2b. Do you have any suggestions as to how these might be handled?

3. Does reporting the data across 59 indicators present any special problems?

**DHO and Assistant DHO**

1. How good has been the technical support of the Ministry of Local Government and/or Ministry of Health in carrying out LQAS in your districts?*

2. Do you have any suggestions on how LQAS results could be utilized more effectively in making decisions?

3. In the last three years, 2012, 2011, and 2010, was the data from your district transmitted to the Ministry of Health or the Ministry of Local Government? If yes, ask for each year if there were any problems?

4. Are you aware of any times that the data from your district has been used by the Ministry of Health or the Ministry of Local Government for planning or making policy? If so, when and for what policy?

* Dropped when we realized that the respondents were talking about STAR E-LQAS.

**Measures of LQAS’s Impact on Services and Challenges Affecting the Impact**

***Reports of Data Collectors***

1. Did you help present the data collected in 2013 to the local health district officer or any other member of the district health management team? Which ones? Where members of the implementing partner present?

2. With the presentation of the data, did you help make recommendations about how to improve the strategies for intervention? In what areas? What recommendations?

3. Did the implementing partner indicate that they would accept your recommendations?

***Reports of Supervisors***

1. Did you help present the data collected in 2013 to the local health district officer or any other member of the district health management team? Which ones? Where members of the implementing partner present?

2. With the presentation of the data, did you help make recommendations about how to improve the strategies for intervention? In what areas? What recommendations?

3. Did the implementing partner indicate that they would accept your recommendations?

Which ones?

***Reports of DHO***

1. In what years did you receive LQAS reports with information on:

a. pre-natal visits

b. births in health clinics or hospitals

2. Have you used LQAS results for making decisions about programme improvement after any of these reports were received ? If yes, in what year and how were they used. If not what are the underlying cases that deter you for not using?

3. Do you have any suggestions on how LQAS results could be utilized more effectively in making decisions?

***Reports of Assistant DHOs***

1. In which years did you receive LQAS reports with information on children being taken to a hospital? [circle answer]

2013 2012 2011 2009

Where recommendations made for changes in district level strategies and tactics made? Which of these were adopted and when?

2. In which years did you receive LQAS reports with information on children being vaccinated?

[circle answer]

2013 2012 2011 2009

Where recommendations made for changes in strategies and tactics? Which of these were adopted and when? Did you find the implementing partner responsive to recommendations from LQAS? If recommendations not adopted, why?

**Organizational Learning and Challenges To Learning**

**DHOs: Safe Motherhood Learning**

1. What was the original strategy of the health care office and of the implementing partner to encourage women to have pre-natal visits?

2. Where there any changes in this plan for pre-natal visits? If so, what were these?

For each change, ask:

a. When? *Specify month and year*

b. Why?

c. What was the actual change?

d. Whom or what was the source of the idea?

3. What was the original strategy of the health care office and of the implementing partner to encourage women to give birth in hospitals or clinics?

4. Where there any changes in this plan for encouraging mothers to give birth in hospitals or clinics? If so, what were these?

For each change, ask:

a. When? *Specify month and year*

b. Why?

c. What was the actual change?

d. Whom or what was the source of the idea?

5. Over the course of the last three years, how often have you meet with other district health officers to discuss strategies for encouraging mothers pre-natal visits and having children in a health facility? How many and which districts were involved? What did you learn in these meetings?

**Assistant DHOs: Child health Learning**

1. What was the original strategy of either the implementing partner or of the local heath district to encourage women to take their children when sick to a hospital or seek assistance:

2. Where there any changes in this plan? If so, what were these?

For each change, ask:

a. When? *Specify month and year*

b. Why?

c. What was the actual change?

d. Whom or what was the source of the idea?

3. What were the strategies of either the implementing partner or of the health district to encourage women to have their children receive vaccines?

4. Where there any changes in this plan? If so, what were these?

For each change, ask:

a. When? *Specify month and year*

b. Why?

c. What was the actual change?

d. Whom or what was the source of the idea?

5. Over the course of the last three years, how often have you meet with other district health officers to discuss strategies for encouraging taking to children to hospitals when sick or to increase the level of immunization? How many and which districts were involved?

What did you learn in these meetings?

**Supervisors and Data Collectors: Additional Learning About LQAS and Services**

1. Over the course of the last three years, how often have you met with [supervisors] [data collectors] from other districts to discuss strategies for data collection, especially about reproductive behavior and child health (pre-natal visits, births in hospitals and clinics, seeking advice for sick children, immunization rates)? If yes, ask How many times? For each event, when? Which districts were involved? What did you learn in these meetings? Where any of these events at the national level?

**Table 1A: Percent of Mothers Who Delivered Their Child in a Health Facility during 2012**

| District | Region | Percent 2012 | Other LQAS Surveys | Also Measured  in 2013 |
| --- | --- | --- | --- | --- |
| Bushenyi | Southwest | 88.4 | 2011, 2010, 2006, 2003 | Yes |
| Mbarara | Southwest | 78.9 | 2011, 2003 | No |
| Kabale | Southwest | 70.5 | 2011, 2010, 2006, 2003 | Yes |
| Kabarole | Western | 80.5 | 2011, 2004 | No |
| Hoima | Western | 63.7 | 2004 | No |
| Kamwenge | Western | 56.8 | 2004 | Yes |
| Mbale | Eastern | 67.7 | 2011, 2010, 2006, 2003 | Yes |
| Kaberamaido | Eastern | 64.2 | 2004 | No |
| Tororo | Eastern | 50.9 | 2011, 2004 | No |

**Table 2A: Scoring Scheme for the Capacity Index and the Control/Coordination Index**

| **Capacity Index Scoring (Training and Experience)** | | | |
| --- | --- | --- | --- |
| ***Health District*** | | | |
| DHO | 1 point for each LQAS survey, maximum 3 points | | |
| Assistant DHO | 1 point for each LQAS survey, maximum 3 points | | |
| Supervisor | 1 point for each LQAS survey, maximum 3 points | | |
| Data Collectors | 1 point for each LQAS survey, maximum 3 points | | |
| **Maximum Index Score** | 12 points | |  |
| **Control/Coordination Index Scoring (Control, Changes, and Planning/Budgeting)** | | | |
| ***Control Scoring*** | **Has Rules Manual** | **Checks on Quality of Data** | |
| Supervisors | 1 | 2 | |
| Data Collectors | 1 | -- | |
| Maximum Sub-Index Score | 4 |  | |
| ***Changes in Interventions ^a^*** | | | |
| DHO Reports | 2 |  | |
| Assistant DHO Reports | 2 |  | |
| Maximum Sub-Index Score | 4 |  | |
| ***Planning and Budgeting ^a^*** |  |  | |
| DHO Reports | 2 |  | |
| Assistant DHO Reports | 2 |  | |
| Maximum Sub-Index Score | 4 |  | |
| **Maximum Index Score** | 12 |  | |
| a. All examples cited by DHO and Assistant DHO were checked against the reports of the data collectors and the supervisors. | | | |

**Table 3A: Number of DHOs, Assistant DHOs (MCH), Supervisors and Data Collectors in Each District Participating in This Study**

| ***District*** | ***DHO*** | ***Assistant DHO*** | ***Supervisors*** | ***Data Collectors*** |
| --- | --- | --- | --- | --- |
| Mbale | Yes | Yes | 5 | 4 |
| Kaberamaido | New** | Not appointed | 6 | 3 |
| Tororo | Yes | Yes | 4 | 5 |
| Kaberole | DHE | No | 1 | 5 |
| Kamwenge | Not appointed | Yes | 2 | 5 |
| Hoima | DHE | New | 2 | 3 |
| Kabale | Yes | Yes* | 4 | 5 |
| Bushenyi | Yes | Yes | 4 | 4 |
| Mbarara | Yes* | Yes | 3 | 4 |
| ***Total*** | ***7*** | ***6*** | ***31*** | ***38*** |

***** telephone interview

** short interview about strategies regarding LQAS

**Table 4A: The Duties of the Data Collectors and Supervisors in Their Districts**

**Sectors**

| ***District*** | ***Health*** | ***Development*** | ***District*** | ***Other**** |
| --- | --- | --- | --- | --- |
| Mbale | 4 | 4 | 0 | 1 |
| Kaberamaido | 2 | 5 | 2 | 0 |
| Tororo | 2 | 6 | 0 | 1 |
| Kabarole | 3 | 3 | 0 | 0 |
| Kamwenge | 5 | 0 | 0 | 2 |
| Hoima | 2 | 1 | 0 | 2 |
| Kabale | 5 | 0 | 2 | 2 |
| Bushenyi | 4 | 2 | 0 | 2 |
| Mbarara | 3 | 2 | 0 | 2 |
| ***Total*** | ***30*** | ***23*** | ***4*** | ***12*** |

* includes parish chief, probation officer, several education officers, a secretary, senior information scientist, sports, etc.
